# PI3K Abrogation Using Pan-PI3K Inhibitor BKM120 Gives Rise to a Significant Anticancer Effect on AML-Derived KG-1 Cells by Inducing Apoptosis and G2/M Arrest

**DOI:** 10.4274/tjh.galenos.2020.2019.0440

**Published:** 2020-08-28

**Authors:** Soroush Sadeghi, Shadi Esmaeili, Atieh Pourbagheri-Sigaroodi, Ava Safaroghli-Azar, Davood Bashash

**Affiliations:** 1 Department of Hematology and Blood Banking, School of Allied Medical Sciences, Shahid Beheshti University of Medical Sciences, Tehran, Iran; 2 Student Research Committee, Department of Hematology and Blood Banking, School of Allied Medical Sciences, Shahid Beheshti University of Medical Sciences, Tehran, Iran

**Keywords:** Acute myeloid leukemia, KG-1 cell, BKM120, PI3K inhibition, NF-κB, c-Myc

## Abstract

**Objective:**

The association between PI3K overexpression and the acquisition of chemoresistance has attracted tremendous attention to this axis as an appealing target to revolutionize the conventional treatment strategies of human cancers. In the present study, we aimed to survey the inhibitory impact of the pan-PI3K inhibitor BKM120 on both cellular and molecular aspects of acute myeloid leukemia (AML)-derived KG-1 and U937 cells.

**Materials and Methods:**

We designed various assays to survey the antitumor impacts and molecular mechanisms underlying the action of BKM120 for the treatment of AML, and we performed experiments to check the effect of BKM120 in combination with idarubicin.

**Results:**

We found that PI3K inhibition diminished cell viability and metabolic activity and exerted a concentration-dependent growth-suppressive effect on the cells. Moreover, we suggested that the ability of BKM120 to induce its antiproliferative properties was mediated through the induction of p21-mediated G2/M cell-cycle arrest. Investigating the effect of inhibitor on the molecular features revealed not only that BKM120 reduced the expression of NF-κB antiapoptotic targets, but also that NF-κB suppression using bortezomib profoundly enhanced the cytotoxicity of the inhibitor, highlighting that the antileukemic effects of BKM120 are mediated, at least partly, through the modulation of the NF-κB pathway. Interestingly, we found that the single agent of BKM120 was unable to significantly alter the expression level of *c-Myc*; however, the capability of BKM120 to reduce the survival rate of AML cells was potentiated upon *c-Myc* inhibition using 10058-F4, suggestive of the plausible contribution of *c-Myc* in leukemic cell response to the PI3K inhibitor.

**Conclusion:**

Taken together, the results of this study reveal the efficacy of BKM120 as a therapeutic approach for AML; however, further investigations should be undertaken to determine the expediency of this inhibitor.

## Abstract (tr)

**Amaç:**

PI3K aşırı ifadesi ile kemoterapiye direnç kazanılması arasındaki ilişki insan kanserlerinin konvansiyonel tedavi stratejilerinde devrim yaratmak yolunda cazip bir hedef oluşturarak bu yolağa olan ilgiyi büyük ölçüde artırmıştır. Bu çalışmamızda pan-PI3K inhibitörü BKM120’nin akut myeloid lösemi (AML) kökenli KG-1 ve U937 hücreleri üzerine hücresel ve moleküler inhibitor etkisini araştırmayı amaçladık.

**Gereç ve Yöntemler:**

AML tedavisinde BKM120’nin etkisinin altında yatan moleküler mekanizmalar ve antitümör tesirini araştırmak için çeşitli yöntemler oluşturduk ve BKM120 idarubisin kombinasyonunun etkisini kontrol etmek için deneyler yaptık.

**Bulgular:**

PI3K inhibisyonunun hücre canlılığı ve metabolik aktiviteyi azalttığını ve hücrelerde konsantrasyona bağımlı olarak büyümeyi baskılayıcı etki gösterdiğini bulduk. Ayrıca, BKM120’nin antiproliferatif etkilerini p21 aracılı G2/M hücre döngüsünü durdurmak yoluyla gösterdiğini ileri sürdük. İnhibitörün moleküler özellikler üzerindeki etkisinin araştırılması sadece BKM120’nin antiapoptotik NF-κB’nin hedeflerinin ifadesini azalttığını değil, fakat aynı zamanda bortezomib kullanarak NF-κB baskılanmasının inhibitörün sitotoksisitesini belirgin artırdığını ortaya koyarak BKM120’nin antilösemik etkisinin en azından kısmen NF-κB yolağı modülasyonuyla olduğunu gösterdi. İlginç olarak, tek başına BKM120’nin *c-Myc* ifadesini anlamlı değiştiremediği, ancak BKM120’nin AML hücrelerinin sağkalım oranlarını azaltma kapasitesinin 10058-F4 aracılı *c-Myc* inhibisyonu ile artması lösemik hücrelerde *c-Myc*’nin PI3K inhibitörüne yanıta katkıda bulunduğunu düşündürmektedir.

**Sonuç:**

Birlikte değerlendirildiğinde bu çalışmanın sonuçları AML için terapötik yaklaşımda BKM120’nin etkililiğini göstermektedir. Ancak bu inhibitörün uygunluğunu belirlemek için ek çalışmalar yapılmalıdır.

## Introduction

Following the comprehension of the important oncogenic pathways that participate in cancer progression, it has become clear that the principal components of these networks will likely act as effective factors in the landscape of anticancer drug design [1]. Among these molecules, phosphatidylinositol 4,5-bisphosphate 3-kinase (PI3K) is currently being explored and the data achieved so far for this protein and its associated network have already translated into novel targeted therapies in the quest for cancer-treatment strategies [2,3]. The recognition that PI3K signaling was aberrantly activated in acute myeloid leukemia (AML) owing to somatic mutations in PIK3Ca, along with the core function of this axis in forcing leukemic cell resistance to apoptosis, paved the way for the recognition of PI3K inhibitors as key elements in the field of AML therapy [4].

Its prominent properties, minor side effects, desirable pharmacokinetic patterns, and broad anticancer impacts on various malignant cells have gained BKM120, a bioavailable specific oral pan-class I PI3K inhibitor, the highest attention among other inhibitors of this pathway [5,6]. In a study conducted by Yang et al. [7], it was illustrated that not only could BKM120 hinder colon cancer cell proliferation through FoxO3a-dependent PUMA induction, but it also potentiated the effect of either 5-fluorouracil or regorafenib on different colon cancer-derived cell lines. Additionally, in vivo investigation revealed that oral administration of BKM120 to cholangiocarcinoma-inoculated nude mice resulted in tumor growth reduction as compared to the control group [8]. The importance of this agent was further revealed in a recent study that demonstrated that abrogation of PI3K using pan-PI3K inhibitor BKM120 decreased the survival of multiple myeloma cells via induction of caspase-3-dependent apoptosis, and it also produced a synergic anticancer effect when combined with carﬁlzomib [9]. While data obtained from these clinical trials and laboratory assays have illustrated the notable impacts of this inhibitor, further surveys are being performed to accurately identify the molecular mechanisms of action of BKM120. Therefore, with the intention of evaluating the potential therapeutic value of pan-PI3K inhibition in AML, we have designed various assays to survey the antitumor impacts and molecular mechanisms underlying the action of BKM120 for treatment of AML. To study the potential therapeutic value of the inhibitor’s cooperation with chemotherapeutic drugs, we also performed experiments to determine the effect of BKM120 in combination with idarubicin, with the end goal of potentiating its effect on AML-derived KG-1 cells.

## Materials and Methods

### Cell Culture

KG-1 and U937 cells were grown in RPMI 1640 medium with 10% fetal bovine serum and 2 mM L-glutamine in a humidified 5% CO_2_ atmosphere at 37 °C. Stock solutions of BKM120, idelalisib, 10058-F4, bortezomib (Selleckchem), idarubicin, and chloroquine (CQ) were provided. AML-derived cells were incubated with the desired concentrations of each agent either alone or in a combined modality. For a negative control, cells were treated with equal concentrations of the solvent.

### Trypan Blue Staining Assay

AML-derived cell lines were treated with either BKM120 alone or co-treatment with the aforementioned inhibitors and the chemotherapeutic drug. After the indicated time duration, trypan blue (Invitrogen) was added to the inhibitor-treated cell mixtures, which were then incubated for approximately 2 min. The numbers of viable cells were calculated using a Neubauer hemocytometer and then the viability index was determined.

### MTT Assay

With the aim of evaluating the antileukemic effects of BKM120, idelalisib, and 10058-F4 on the AML-derived cells, the MTT assay was applied. The cells (5x10^3^) were seeded in 96-well plates in the presence of the inhibitors and incubated in a humidified 5% CO_2_ incubator at 37 °C. At different time intervals, the MTT solution 5 mg/mL in phosphate buffered saline (PBS)] was added to each well and incubated for 3 h at 37 °C. After discarding the media, the percentage of the metabolic activity of cells was evaluated by dividing the optical density (OD) of resulting formazan measured by an enzyme-linked immunosorbent assay (ELISA) reader in the drug-treated groups by the OD of the control group.

### Acridine Orange Staining Assay

To investigate the contributory role of autophagy in BKM120 cytotoxicity on KG-1, the cells were treated with increasing concentrations of the autophagy inhibitor CQ (Sigma). The cells were then washed with PBS and dyed with 1 µg/mL acridine orange (Merck) for 15 min in the dark. The differences in acidity of autophagic lysosomes and the cytoplasm/nucleolus were visualized under a fluorescence microscope and by flow cytometry (BD FACSCalibur, BD Biosciences, San Jose, CA, USA).

### BrdU Cell Proliferation Assay

To assess the inhibitory effects of BKM120 on DNA synthesis, the 5-bromo-2-deoxyuridine cell proliferation assay was performed using an ELISA kit (Roche, Mannheim, Germany) based on the manufacturer’s guidelines [10].

### Real-Time PCR (RNA Extraction and cDNA Synthesis)

In order to extract total RNA from the KG-1 cell line, the High Pure RNA Isolation Kit (Roche, Mannheim, Germany) was used and results were quantified spectrophotometrically with a NanoDrop device (NanoDrop Technologies, Wilmington, DE, USA). Afterwards, a cDNA synthesis kit (Takara Bio, Shiga, Japan) was used to perform reverse transcription reactions. The cDNA was subjected to quantitative real-time PCR (qRT-PCR) and then fold change values were calculated based on the 2^-ΔΔct^ relative expression formula. *ABL* was amplified as a housekeeping gene. The sequences of primers used in the experiment are summarized in Table 1.

### Flow Cytometric Analysis of Apoptosis and Cell Cycle

The effects of BKM120 on the programmed cell death and progression of the cell cycle were evaluated by annexin-V staining assay in AML-derived cells. BKM120-treated cells were harvested after 48 h of treatment, washed with PBS, and subjected to flow cytometric analysis of annexin-V and PI staining assays as described in our previous study [11].

### Statistical Analysis

Our data are presented as the mean ± standard deviation (SD) (p<0.05) of three individual experiments; all experiments were carried out in triplicate. Differences among experimental variables were determined by the use of Student’s t-test, employing SPSS and dne-way analysis of variance.

## Results

### Cytotoxic Effects of PI3K Inhibitors on AML-Derived KG-1 Cells

**Figure 1:**
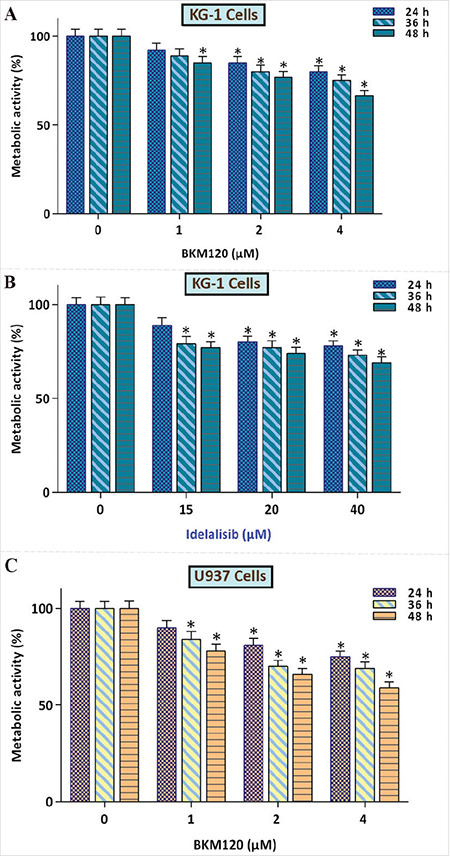
Cytotoxic effect of PI3K inhibitors BKM120 on AML-derived cells. A and B) Suppression of the PI3K signaling pathway using BKM120 and idelalisib reduced the metabolic activity of KG-1 cells. C) In agreement with the results for KG-1, BKM120 also decreased the survival of U937 cells. Values are given as mean ± SD of three independent experiments. *: p≤0.05 represents significant changes from the untreated control. ANOVA was used to compare the mean of the metabolic activity tests. SD: Standard deviation, AML: acute myloid leukemia.

To investigate the cytotoxic effects of the pan-PI3K inhibitor BKM120 on AML, KG-1 cells were incubated with various concentrations of the inhibitor, and then the MTT assay was applied. Our results suggested that upon PI3K inhibition the metabolic activity of the KG-1 cell line was diminished in a concentration-dependent manner (Figure 1A). To validate the cytotoxic effects of PI3K inhibition on this cell line, we also assessed the effects of idelalisib, a highly selective inhibitor of the PI3K p110-δ isoform. In agreement with the results obtained for BKM120, it was evident that exposure of the cells to idelalisib resulted in a clear decline in the survival rate of the cells (Figure 1B). Our supplementary experiments investigating the effects of PI3K inhibition on AML further confirmed the cytotoxic effect of BKM120 on AML-derived U937 cells (Figure 1C).

### Antiproliferative Effects of BKM120 on KG-1 Cells Are Mediated Through the Induction of G2/M Arrest

**Figure 2:**
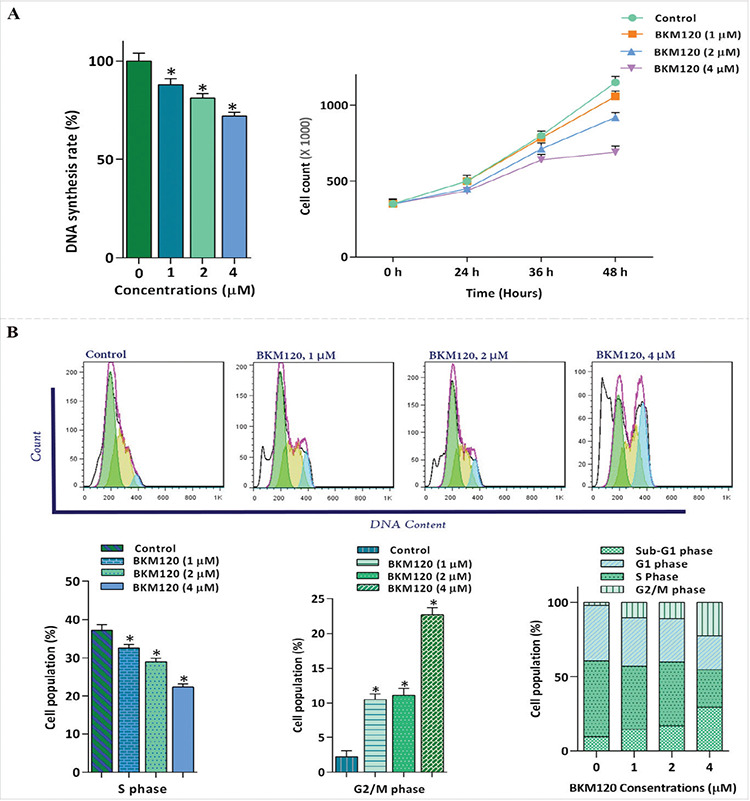
BKM120’s effect on the proliferative capacity of KG-1 cells. A) The ability of BKM120 to blunt the capability of the cells to replicate their DNA and to proliferate was assessed using BrdU and trypan blue exclusion assays, respectively. B) Evaluating the effect of the agent on the distribution of cells in different phases of the cell cycle revealed that BKM120 halted the transition of cells from the G2/M phase as well as reducing the percentage of cells in the S phase of the cell cycle after 48 h of treatment. Values are given as mean ± SD of three independent experiments. *: p≤0.05 represents significant changes from the untreated control. ANOVA was used for statistical analysis. SD: Standard deviation.

Multiple lines of evidence have revealed that the PI3K signaling pathway is tightly associated with a diverse group of cellular functions, including cell proliferation and DNA synthesis [12]. Our results demonstrated that the treatment of the cells with this pan-PI3K inhibitor not only decreased the DNA synthesis rate but also inhibited the proliferative capacity of the cells, as revealed by the reduction in the number of viable cells (Figure 2A). Moreover, the cell population in the S phase was decreased from 37.2% in the control group to 22.4% in the 4 µM-treated cells (Figure 2B). Meanwhile, the percentage of cells in the G2/M phase of the cell cycle grew by nearly 10-fold in comparison with the control group, suggesting that the antiproliferative effects of the agent on KG-1 are mediated, at least partly, by the induction of G2/M arrest.

### Stimulatory Effect of *c-Myc* Suppression on BKM120-Induced Cytotoxicity in KG-1 Cells

**Figure 3:**
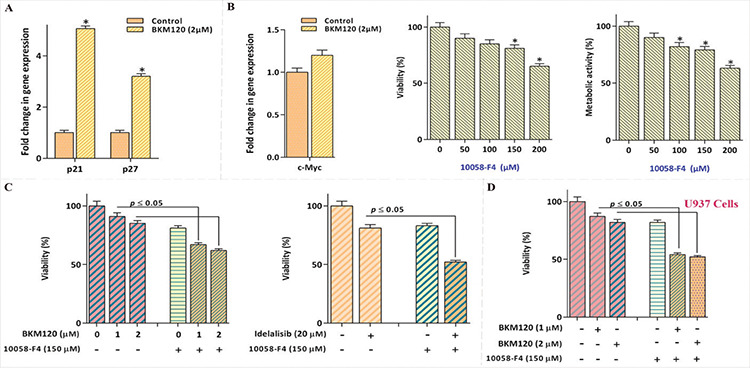
*c-Myc* abrogation potentiated the cytotoxic effect of both BKM120 and idelalisib in KG-1 and U937 cells. A) Treatment of KG-1 cells with BKM120 escalated the expression levels of p21 and p27. B) Although BKM120 could not significantly alter the mRNA expression level of *c-Myc*, its inhibition using 10058-F4 decreased the viability and metabolic activity of KG-1 cells (the significance of the results was evaluated by the use of ANOVA). The endogenous gene used in the experiments was ABL. Values are given as mean ± SD of three independent experiments. *: p≤0.05 represents significant changes from the untreated control. C) Upon 24 h of exposure to the *c-Myc* inhibitor, the antileukemic effects of both PI3K inhibitors were reinforced in AML-derived cell lines. D) Synergistic effect of BKM120 and 10058-F4 on U937 cells indicated the same result. The t-test was used to determine the statistical significance of the results of qRT-PCR analysis as well as synergistic experiments. SD: Standard deviation, RT-PCR: reverse transcription-polymerase chain reaction.

Growing numbers of experiments reveal that, upon PI3K signaling*, c-Myc *is activated and subsequently drives the cell cycle through the suppression of p21 and p27 [13,14]. We found that BKM120 upregulated the mRNA expressions of both p21 and p27; however, we could find no significant alteration in *c-Myc *level (Figure 3A). It has been reported that the unrestrained activation of *c-Myc* could be directly responsible for the lower sensitivity of malignant cells to PI3K inhibitors [15]. Accordingly, we found that the suppression of *c-Myc* using 10058-F4 not only reduced KG-1 cell survival (Figure 3B) but also boosted the cytotoxic effects of either BKM120 or idelalisib when used in a combined-modality treatment (Figure 3C). Complementary investigations also confirmed that co-treatment of U937 cells with BKM120 and 10058-F4 resulted in superior cytotoxicity as compared to each drug alone (Figure 3D).

### BKM120 Induced Apoptotic Cell Death in AML-Derived KG-1 Cells

**Figure 4:**
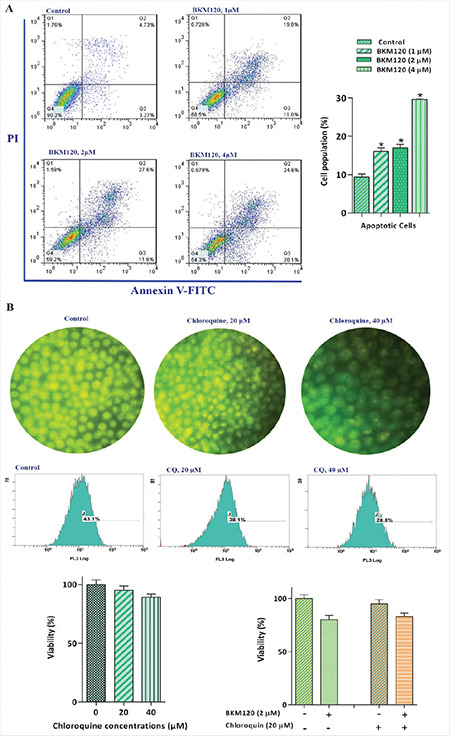
BKM120 exerted its cytotoxic effect partly through induction of apoptosis. A) The percentage of annexin-V-positive inhibitor-treated cells was elevated after 48 h of treatment with BKM120 as compared to the untreated group. The ANOVA test was used to determine the statistical significance of the results of flow cytometry analysis. B) The inhibition of autophagy, as represented by a reduction of fluorescence intensity, could not induce a significant stimulatory effect on BKM120’s anticancer effect. Values are given as mean ± SD of three independent experiments. *: p≤0.05 represents significant changes from the untreated control. SD: Standard deviation.

The PI3K pathway affects a notable array of intracellular events that directly or indirectly influence whether a cell will undergo apoptosis [16,17,18]. Our results showed that the amounts of annexin-V cells were elevated in comparison with the control group, which is in agreement with the augmented cells in the sub-G1 phase (Figure 4A). Numerous studies have demonstrated that the alteration of the autophagy system may act as an important contributory mechanism affecting the extent of cell death induced by anticancer agents in malignant cells [19,20,21]. To investigate the contributory role of autophagy in BKM120 cytotoxicity, the effect of CQ, a well-known autophagy inhibitor, was examined as both a single agent and in combination with the PI3K inhibitor. Our results showed that the inhibition of autophagy, as revealed by a concentration-dependent reduction in the fluorescence intensity ratio detected by either microscopic imaging or flow cytometric analysis, did not induce a stimulatory effect on BKM120’s anticancer effect (Figure 4B).

### Alteration of Apoptosis-Related Genes Upon Treatment of KG-1 Cells with BKM120

**Figure 5:**
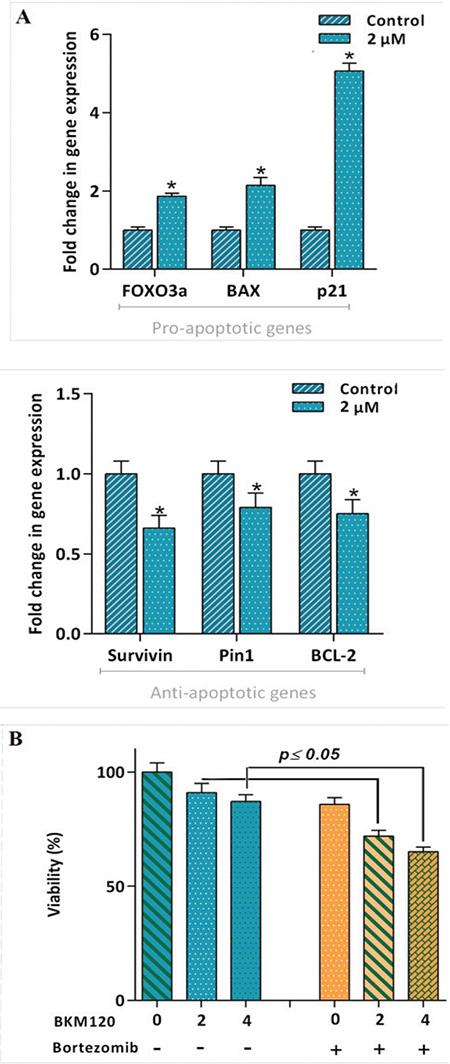
BKM120 altered the expression of apoptosis-related genes. A) After 48 h of treatment of the cells with BKM120 at a concentration of 2 μM, the expression levels of pro- and antiapoptotic genes were determined using qRT-PCR. The inhibitor could not only increase the expression of proapoptotic genes but could also diminish the mRNA expression levels of antiapoptotic genes.* ABL* was used as an endogenous gene. B) Proteasome inhibitor bortezomib potentiated the effect of BKM120 in KG-1 cells. Values are given as mean ± SD of three independent experiments. *: p≤0.05 represents significant changes from the untreated control. The t-test was used to determine the statistical significance of the results of qRT-PCR analysis as well as synergistic experiments. SD: Standard deviation, RT-PCR: reverse transcription-polymerase chain reaction.

Multiple line of evidence revealed that a fine-tuned balance between pro- and antiapoptotic target genes determines the receptiveness of cancer cells to death stimuli [22,23]. Our results showed that BKM120 upregulated the mRNA expressions of proapoptotic genes and diminished the transcription of antiapoptotic target genes of NF-κB (Figure 5A). Based on our findings and with reference to the considerable studies pointing to the tight crosstalk between NF-κB and the PI3K pathway [24], we examined the effect of NF-κB suppression on the anticancer impact of the inhibitor. Our results showed that the suppression of NF-κB using a well-known proteasome inhibitor, bortezomib, amplified the repressive effect of the PI3K inhibition on KG-1 cell survival (Figure 5B).

### Synergistic Effect of BKM120 with Idarubicin in AML-Derived KG-1 Cells

**Figure 6:**
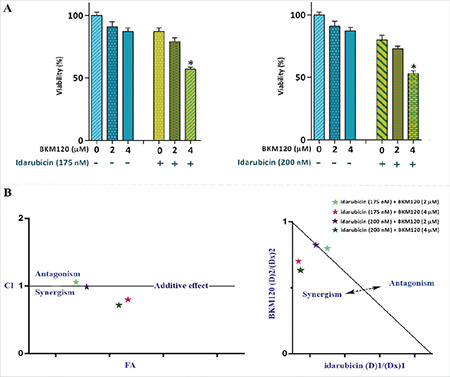
BKM120 and idarubicin co-treatment resulted in superior cytotoxicity in KG-1 cells. A) BKM120 could potentiate the antileukemic effect of idarubicin on AML-derived cell lines. B) The results of both the combination index (CI) and isobologram highlight the synergistic effect between BKM120 and idarubicin. Values are given as mean ± SD of three independent experiments. *: p≤0.05 represents significant changes from the untreated control. The t-test was used to determine the statistical significance of the results of synergistic experiments. SD: Standard deviation, AML: acute myloid leukemia.

As shown in Figure 6A, the combination of BKM120 at a concentration of 4 µM with idarubicin (175 and 200 nM) was more effective in inhibiting cell growth and survival as compared to either drug alone. To test whether the interactions between these drugs were synergistic or caused by additive effect, the combination index (CI) and isobologram were calculated. Determination of both CI and dose reduction index (DRI) values clarified that, unlike the lower concentration of BKM120 (2 µM), the higher concentration of this agent has the ability to boost the cytotoxic and antiproliferative effects of this well-known chemotherapeutic drug used in AML treatment protocols (Figure 6B). Values of CI and DRI achieved after 24 h of treatment of KG-1 cells are summarized in Table 2.

## Discussion

With the increasing comprehension of the molecular mechanisms underlying human cancers and the recent achievements in understanding the events connected to tumor cells’ responses to chemotherapeutic drugs, cancer treatment procedures have changed substantially [25]. Adequate laboratory studies demonstrated that the overactivation of the PI3K network is not only associated with cancer progression, but also results in the acquisition of a chemoresistance phenotype [26,27]. It has also been reported that the aberrant activation of PI3K is coupled with poor prognosis in a variety of human malignancies, including AML [28]. Our data have revealed that the suppression of PI3K using BKM120 could remarkably reduce both the survival and the metabolic activity of the AML-derived KG-1 and U937 cell lines, which is in agreement with the results reported by Allegretti et al. [29], who showed a favorable antileukemic effect of the inhibitor against AML cells but not the normal counterpart. In addition, Pillinger et al. [30] revealed that PI3K inhibition using three PI3K inhibitors, LY294002, IPI-145, and CAL-101, significantly reduced the survival of AML cells in vitro or in vivo. In harmony with the results for BKM120, treatment with the isoform-specific PI3Kδ inhibitor idelalisib further confirmed the cytotoxic effects of PI3K inhibition in KG-1 and U937 cells. Additionally, PI3K suppression potentially reinforced the antileukemic property of a commonly used chemotherapeutic drug in AML treatment, idarubicin, highlighting the promising effect of the inhibitor either as a single agent or in combined-modality treatment.

In light of the growth-suppressive effects of PI3K inhibitors on tumor cells and based on the central role of this network in the progression of the cell cycle [31], the effect of the agent on cell distribution was examined using flow cytometric analysis. Our results demonstrated that the treatment of KG-1 with BKM120 not only inhibited proliferative capacity by reducing DNA replication and the number of viable cells but also led to an increased percentage of cells in the G2/M phase, suggesting that the antiproliferative effects of the inhibitor are mediated, at least partially, through the induction of G2/M arrest. In an investigation of the effects of PI3K suppression on multiple myeloma cells, the inhibitory effect of pan-PI3K inhibition on the survival of both KMM-1 and RPMI 8226 cells via the induction of SIRT1-mediated G2/M arrest was also highlighted [9]. Additionally, Xie et al. [32] showed that puquitinib, an orally available PI3Kδ inhibitor, hindered the proliferative capacity of AML-derived cell lines through induction of G1 cell-cycle arrest. Among examples of overactivation of malignant signaling networks, and foremost of the PI3K pathway, the *c-Myc *oncogene is explicitly activated and subsequently leads to cell cycle progression via the inhibition of cell cycle-related genes such as p21 and p27 cyclin-dependent kinase inhibitors [33]. Remarkably, while BKM120-induced G2/M arrest was associated with the upregulation of p21 and p27 expression, we could find no noticeable alteration in *c-Myc* mRNA levels, suggesting the probable contribution of the *c-Myc* oncogene with less sensitivity of leukemic cells to the PI3K inhibitors. Accordingly, investigation of the effects of the small-molecule inhibitor of *c-Myc* revealed that 10058-F4 reduced KG-1 cell survival and sensitized the cells to lower concentrations of either BKM120 or idelalisib, supporting our hypothesis that the *c-Myc* inhibitors may restore leukemic cell sensitivity to PI3K inhibitors when administered as part of combination regimens.

**Figure 7:**
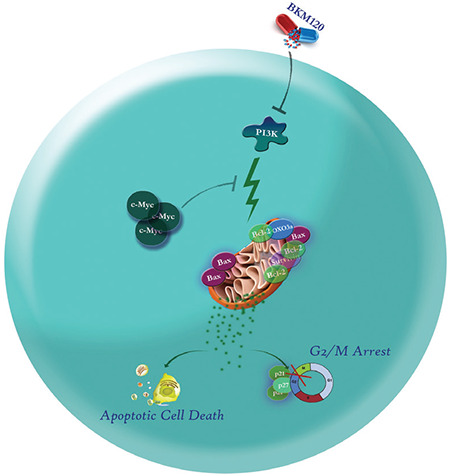
Schematic representation suggested for the plausible mechanisms of action of BKM120 in KG-1 cells. Via abrogation of the PI3K signaling pathway, BKM120 diminished the survival and proliferative capacity of the cells, possibly through escalation of cyclin-dependent kinase inhibitors p21 and p27 and subsequent G2/M arrest. As presented, suppression of PI3K induced apoptotic cell death through upregulation of proapoptotic genes and downregulation of antiapoptotic genes, which may, at least in part, be overshadowed by the activation of *c-Myc* expression.

Extensive experiments have revealed an alternative mechanism through which p21 could induce apoptotic cell death through modulation of the NF-κB pathway [34]. Moreover, the contributing role of NF-κB in the mechanism of action of several chemotherapeutic drugs has been clearly established in several reports [35]. Our results showed that the antileukemic property of BKM120 was not only remarkably coupled with the reduction in the expression levels of NF-κB antiapoptotic target genes; additionally, NF-κB suppression using the well-known proteasome inhibitor bortezomib could profoundly enhance the cytotoxic effect of BKM120 on KG-1 cells, substantiating the claim that the antileukemic effects of the inhibitor are mediated, at least partly, through modulation of the NF-κB pathway. The results of previous studies suggested that NF-κB could provide a signal that in turn could activate the autophagy system in cancerous cells and thereby attenuate death signals [36]. Of particular interest, we found that the antitumor effect of BKM120 was not influenced by the inhibition of autophagy and, as far as we are aware, our results suggest for the first that the induction of cell death in KG-1 cells is probably mediated independently of the autophagy system (Figure 7).

## Conclusion

Taken together, the results of the present study show that BKM120 is a favorable anticancer agent, either as a single agent or in a combined-modality strategy in cancer therapeutics; however, further investigations should be undertaken to determine the suitability of the inhibitor, in particular for the treatment of AML.

## Figures and Tables

**Table 1:**
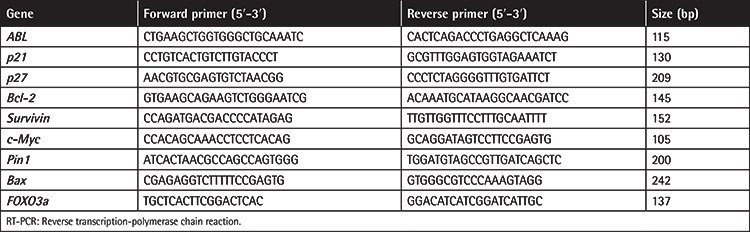
Nucleotide sequences of primers used for real-time RT-PCR.

**Table 2:**
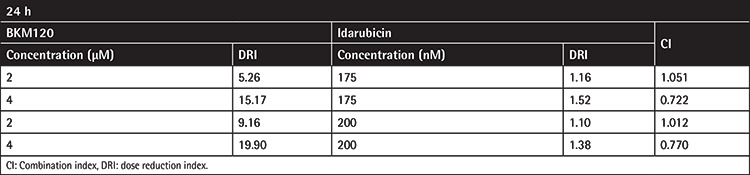
Combination index and dose reduction index for the drug combination of BKM120 and idarubicin.
